# Impact of Beta-Blocker Therapy on Pregnant Women With Long QT Syndrome: A Systematic Review

**DOI:** 10.7759/cureus.71678

**Published:** 2024-10-17

**Authors:** Bassel Alrabadi, Hadeel A Al Kayed, Nour Alshujaieh, Ahmad Saadeh, Ro'ya Khanfar

**Affiliations:** 1 Internal Medicine, Al-Balqa Applied University, Al-Salt, JOR; 2 Medicine and Surgery, The University of Jordan, Amman, JOR; 3 Internal Medicine, The University of Jordan, Amman, JOR; 4 Obstetrics and Gynaecology, The University of Jordan, Amman, JOR; 5 Obstetrics and Gynaecology, An-Najah National University, Nablus, PSE

**Keywords:** arrhythmia, beta-blockers, intrauterine growth restriction, long qt syndrome, pregnancy

## Abstract

Long QT syndrome (LQTS) presents significant challenges for pregnant women due to the elevated risk of arrhythmic events. Beta-blocker therapy is a cornerstone of treatment in managing LQTS, but its use during pregnancy introduces potential fetal risks. This systematic review synthesizes the evidence on the efficacy and safety of beta-blocker therapy in pregnant women with LQTS. A systematic review was conducted to assess studies on beta-blocker use in pregnant women diagnosed with LQTS. We reviewed observational studies and case reports to evaluate maternal cardiac outcomes, fetal safety, and the need for postpartum management. Studies were selected based on inclusion criteria focused on LQTS, beta-blocker therapy during pregnancy, and associated maternal and fetal outcomes.

Our review showed that beta-blocker therapy significantly reduced arrhythmic events in pregnant women with LQTS, particularly in symptomatic carriers. Multiple studies demonstrated that the continuation of beta-blocker therapy throughout pregnancy and the postpartum period minimized maternal cardiac risks. However, beta-blocker use was associated with fetal risks such as intrauterine growth restriction (IUGR), lower birth weight, and, in some cases, bradycardia. Atenolol, in particular, was linked to significant fetal growth restrictions, while other beta-blockers, such as propranolol and metoprolol, showed more variable effects on fetal outcomes. Despite these concerns, some studies reported favorable postnatal growth recovery in infants exposed to beta-blockers in utero.

Beta-blocker use must be carefully managed due to potential fetal risks. Regular monitoring, individualized treatment plans, and appropriate selection of beta-blocker type are essential to optimizing both maternal and fetal outcomes. Further research is necessary to refine treatment protocols and reduce fetal risks associated with beta-blocker therapy during pregnancy.

## Introduction and background

Long QT syndrome (LQTS) is an inherited cardiac channelopathy characterized by a prolonged QT interval on electrocardiography (ECG), which disrupts normal ventricular repolarization. This condition significantly increases the risk of polymorphic ventricular tachycardia, such as torsades de pointes, and sudden cardiac death (SCD), even in individuals with structurally normal hearts. LQTS affects approximately 1 in 2,000 individuals, with the majority of cases (~90%) being inherited, underscoring the critical role of genetic factors in its development [1,2].

In women with LQTS, pregnancy introduces a unique set of challenges, as changes in sex hormone levels may modulate cardiac repolarization [3]. Notably, the risk of arrhythmia tends to decrease during pregnancy but rises considerably in the postpartum period, with the risk of arrhythmias being up to four times higher than prior to pregnancy [3,4]. Estrogen and progesterone, the primary hormones involved, are key contributors to these shifts in arrhythmia risk. Estrogen exhibits antiarrhythmic properties, while progesterone, which is markedly elevated during pregnancy and declines postpartum, reduces the risk of arrhythmias [5]. In contrast, hormones such as oxytocin and prolactin, which become more prominent in the postpartum period, act as pro-arrhythmic modifiers [6]. Additionally, the risk of arrhythmia differs according to the LQTS genotype, with a significant increase noted in individuals with LQTS type 2 (LQT2) [4].

For women with inherited arrhythmia syndromes, such as LQTS, it is essential to have a multidisciplinary team specializing in pregnancy, cardiac arrhythmias, and genetics to conduct thorough risk assessments and provide pre-pregnancy care [7]. The management of LQTS in these women involves various strategies, with beta-blockers being a cornerstone therapy due to their significant ability to reduce the risk of life-threatening arrhythmias [8]. According to the 2017 guidelines from the American Heart Association (AHA), the American College of Cardiology (ACC), and the Heart Rhythm Society (HRS) on ventricular arrhythmias (VA) and SCD, as well as the 2018 European Society of Cardiology (ESC) guidelines for the management of cardiovascular diseases during pregnancy, beta-blockers are strongly recommended for women with congenital LQTS during pregnancy and the postpartum period (Class I, level of evidence C) [8,9].

Despite the clear benefits of beta-blocker therapy in reducing arrhythmic risk, concerns about its safety and effectiveness in pregnant women with LQTS, as well as its impact on the fetus, remain. There are ongoing concerns that beta-blocker therapy may increase the risk of intrauterine growth restriction (IUGR), fetal bradycardia, and congenital malformations [10,11]. Consequently, it is critical to individualize treatment and implement strategies for continuous maternal and fetal monitoring to track treatment efficacy and adjust dosages accordingly.

In this narrative review, we aim to evaluate the effectiveness of beta-blockers in preventing arrhythmias and sudden cardiac events during pregnancy and the postpartum period while also examining the potential risks associated with beta-blocker use during pregnancy.

## Review

Methodology

Study Design and Eligibility Criteria

We adhered strictly to the guidelines outlined in the Preferred Reporting Items for Systematic Reviews and Meta-Analyses (PRISMA) statement throughout the synthesis of this review [12]. All primary study designs were considered eligible for inclusion. The inclusion criteria for this review consisted of (a) studies that examine the efficacy of beta-blocker therapy in reducing arrhythmic events in pregnant women with LQTS; (b) studies that evaluate the safety and risks of beta-blocker therapy for the fetus during pregnancy; (c) research that reviews current clinical guidelines and recommendations for the use of beta-blockers in pregnant women with LQTS; (d) studies that outline monitoring and management strategies for maternal and fetal well-being during beta-blocker therapy; and (e) research that addresses postpartum management of LQTS in women treated with beta-blockers. Exclusion criteria included (a) studies with inadequate data on maternal or fetal outcomes; (b) studies published in languages other than English; and (c) studies where the full text was unavailable.

Search Strategy

We conducted a comprehensive search of the following electronic databases: PubMed, Google Scholar, and Scopus. The search terms used included: "beta-blocker therapy," "long QT syndrome," "pregnancy," "arrhythmias," and "fetal safety." Only articles written in English were eligible for inclusion. The search strategy used for each database is provided in Table 1.

**Table 1:** Search strategy

Database	Search strategy	Applied filters
Google Scholar	"beta blocker" AND (intitle:mother OR intitle:pregnant OR intitle:pregnancy OR intitle: postpartum) AND (intitle:"QT interval" OR intitle:"long QT")	No filters
Scopus	(TITLE-ABS-KEY (beta AND blocker*) AND TITLE-ABS-KEY (mother* OR pregnant OR pregnancy OR postpartum) AND TITLE-ABS-KEY (qt AND interval OR long AND qt))	No filters
PubMed	Search: ((#8) AND (#7)) AND (#6) #8: (beta blocker*[Text Word]) OR (management[Text Word]) #7: ((mother*[Title]) OR (pregnant[Title])) OR (pregnancy[Title] OR (postpartum)) #6: (QT interval[Title]) OR (long QT[Title])	Full text, English

Study Selection

**Figure 1:**
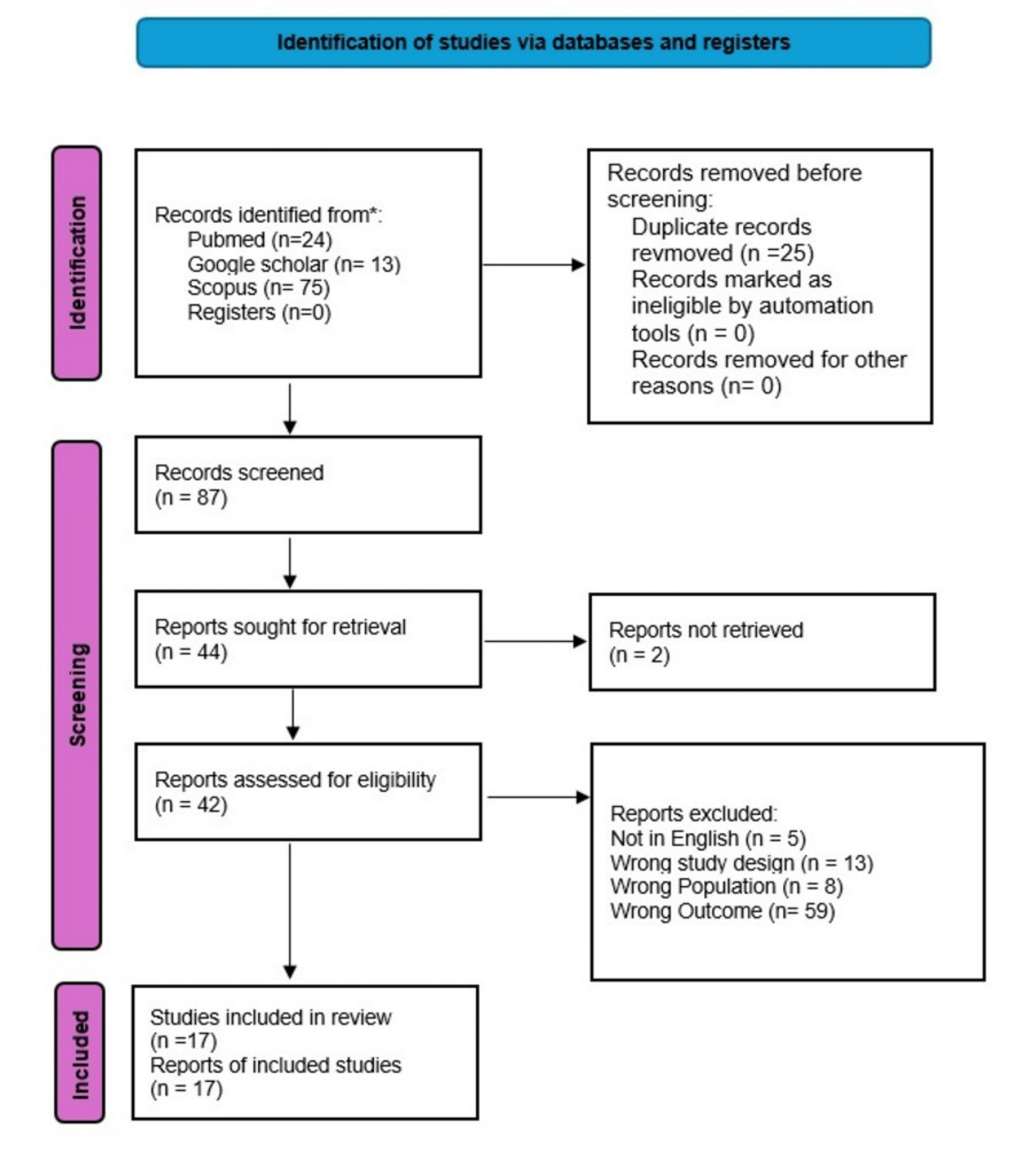
PRISMA flow chart for selection of studies PRISMA: Preferred Reporting Items for Systematic Reviews and Meta-Analyses

The initial search identified multiple records. After the removal of 25 duplicates, 87 unique articles remained. Two independent reviewers screened the titles and abstracts, which resulted in 44 studies being deemed eligible for full-text review to assess compliance with the inclusion criteria outlined above. Disagreements between reviewers were resolved through discussion. Following a thorough evaluation, 17 studies were included in the systematic review. Figure 1 presents the PRISMA flow chart, depicting the study selection process.

Data Extraction

Two independent reviewers extracted data from the eligible studies using pre-defined standardized data extraction forms. These forms were developed based on established guidelines for systematic reviews and tailored to capture the key information necessary for this review. The forms included fields to record study characteristics (e.g., study design, sample size, setting, patient demographics), intervention details (e.g., type of beta-blocker used, dosage, duration), and outcomes (e.g., maternal and fetal outcomes, arrhythmic events). The forms were piloted on a small sample of studies (five studies) to ensure clarity and consistency in data collection. Any ambiguities were resolved during this pilot phase, and the forms were revised accordingly to improve usability and accuracy. Discrepancies between the two reviewers were resolved through discussion, and a third reviewer was consulted if consensus could not be reached. A final version of the data extraction forms was used throughout the study to ensure consistency in data collection.

Data Management and Storage

The data extracted from the eligible studies were securely stored in a shared digital file accessible only to the authors of this review. The extracted data were organized using standardized forms, and any updates or modifications were tracked to ensure accuracy. Regular backups were created to prevent data loss. All study data will be retained for future reference and transparency in accordance with institutional data retention policies.

Outcomes and Measures

The primary outcome was the efficacy of beta-blocker therapy, measured by the incidence and severity of clinically significant arrhythmic events as determined by ECG findings and clinical symptoms during pregnancy and the postpartum period. The secondary outcomes were categorized into four domains: (a) Safety: fetal outcomes, including bradycardia, IUGR, and neonatal complications; (b) Clinical Guidelines: recommendations for beta-blocker therapy across different stages of pregnancy and the postpartum period; (c) Monitoring Strategies: methods for assessing maternal and fetal well-being; and (d) Postpartum Management: continued beta-blocker therapy and follow-up strategies during the postpartum phase.

Quality and Risk of Bias Assessment

We conducted a comprehensive methodological quality assessment to ensure the reliability of the evidence included in our systematic review. Cohort and case-control studies were assessed with the Newcastle-Ottawa Scale (NOS), while case reports were reviewed based on a checklist developed from the CARE guidelines [13].

The risk of bias assessment was carried out independently by two authors. In cases where discrepancies arose, these were initially resolved through discussion between the two reviewers. If consensus could not be reached, a third independent reviewer was consulted to adjudicate, ensuring that the final assessment minimized subjectivity and bias. Further details regarding the specific criteria used for the assessment of bias, along with the full quality assessment process and the results of the assessment, can be found in the Appendices. The registered PROSPERO ID for this study is CRD42024563355.

Results

The database search yielded 112 potentially relevant studies. After removing duplicates, 87 studies remained. Following a review of the titles and abstracts, 43 studies were excluded. The remaining 42 full-text studies were assessed for eligibility, and ultimately, 17 studies met the inclusion criteria and were included in the analysis.

The review includes nine observational studies and eight case reports. The primary objective was to evaluate maternal and neonatal outcomes associated with beta-blocker use during pregnancy. Across these studies, a variety of beta-blockers, including propranolol, atenolol, metoprolol, nadolol, and carvedilol, were assessed for their safety and efficacy. A summary of the key characteristics and outcomes of the included studies is presented in Table 2.

**Table 2:** Characteristics and key outcomes of included studies IUGR: Intrauterine growth restriction; LQTS: Long QT syndrome; ICD: Implantable cardioverter-defibrillator; SGA: Small gestational age; TdP: Torsades de pointes; LVEF: Left ventricular ejection fraction; PVCs: Premature ventricular contractions; VF: Ventricular fibrillation

Study references	Authors	Design	Sample size	Intervention	Outcome
[4]	Seth et al.	Multi-Center Retrospective Cohort Study	N = 391	Did not specify	Women with long QT syndrome (LQTS), especially those with LQT2, have higher postpartum risks. Beta-blockers reduce annual events from 3.7 to 0.8. The risk peaks in the first 6 months postpartum and decreases thereafter.
[7]	Regitz-Zagrosek et al.	Case Report	N = 2	Bisoprolol; Metoprolol 50 mg/day	LQTS type 2 patients have increased postpartum cardiac events. ECGs should be performed every 1-2 weeks after delivery, with monitoring if QT exceeds 500 msec.
[8]	Ishibashi et al.	Multi-Center Retrospective Cohort Study	N = 76	Propranolol; 20-60 mg/day; Atenolol 50 mg/day; Bisoprolol 5 mg/day; Carteolol 15 mg/day	The beta-blocker group had lower birth weights and more preterm births. However, there were no significant differences in cardiac events or malformations, and beta-blockers did not harm fetal growth. Early beta-blocker use is crucial for managing cardiac events.
[10]	Ersbøll et al.	Single-Center Retrospective Cohort Study	N = 802	Labetalol; Carvedilol; Sotalol; Propranolol; Pindolol; Atenolol; Metoprolol	No stillbirths occurred. Group A had smaller gestational age (SGA) infants and a greater birthweight deviation than Group B. Longer beta-blocker use reduced birthweight deviation, while oral beta-blockers increased the risk of SGA.
[14]	Heradien et al.	Case-Control Study	N = 36	Did not specify	Women with LQTS, particularly those with LQT2, face increased postpartum risks. Beta-blockers significantly lower the annual event rate, with the risk of cardiac events peaking within 6 months after birth.
[15]	Kaizer et al.	Multi-Center Retrospective Cohort Study	N = 267	Nadolol; Propranolol Metoprolol; Atenolol 100 mg/day	Nadolol, propranolol, and metoprolol did not harm fetal heart rates but caused bradycardia in LQT1 and LQT2 fetuses. Atenolol is not recommended due to fetal growth restriction. However, beta-blocker therapy (nadolol, propranolol, metoprolol) should be continued for pregnant LQTS patients to reduce cardiac risks.
[16]	Kamiya et al.	Case Report	N = 1	Verapamil 120 mg/day; Atenolol 25 mg/day	With maternal beta-blocker use, the fetus grew normally for its gestational age. Labor started spontaneously at 39 weeks without complications, suggesting beta-blockers may benefit Andersen-Tawil syndrome and other LQTS types.
[17]	Tanaka et al.	Single-Center Retrospective Cohort Study	N = 25	Propranolol; Bisoprolol; Atenolol; Verapamil	Beta-blocker use may have helped prevent cardiovascular events during labor.
[18]	Kubo et al.	Case-Control	N = 1	Landiolol 57.6 mg/day; Ephedrine	Delivery was smooth, with no complications. The fetus had typical vital signs and Apgar scores. Postoperatively, propranolol was administered, and recovery was uneventful.
[19]	Albertini et al.	Single-Center Retrospective Cohort Study	N = 23	Bisoprolol; Metoprolol; Atenolol; Nadolol; Propranolol 120 mg/day	Beta-blocker use led to 1 preterm birth and 9 term births, with term babies having lower birth weights. Additionally, atenolol and nadolol increased the risk of intrauterine growth restriction (IUGR). Maternal LQTS and beta-blocker use affect fetal outcomes.
[20]	Lee et al.	Case Report	N = 1	Nadolol; Midodrine; Mexiletine 150 mg/day; Propranolol 60 mg/day	Beta-blocker use may have helped prevent cardiovascular events during labor.
[21]	Milner et al.	Case Report	N = 1	Nadolol 60mg/day with IV magnesium	Cesarean section at 35 weeks was performed due to growth restriction and preeclampsia. Postpartum, an implantable cardioverter-defibrillator (ICD) was placed for a QTc >500 ms. The infant experienced Torsades de pointes (TdP) post-delivery, treated with propranolol, lidocaine, and mexiletine, maintaining a normal rhythm with monitoring and defibrillator use.
[22]	Huttunen et al.	Single-Center Retrospective Cohort Study	N = 104	Did not specify	LQT1 patients exposed to maternal beta-blockers were smaller at birth but showed catch-up growth. Additionally, maternal beta-blocker use was associated with lower cord thyroid-stimulating hormone (TSH) levels.
[23]	Papantoniou et al.	Case Report	N = 1	Did not specify	Regular high-risk visits and ultrasounds were conducted; beta-blockers were used despite growth risks. An elective cesarean was performed at 36 weeks with a smooth recovery and ECG monitoring. The female infant weighed 2800 g with Apgar scores of 9/10, regular cardiac checks, and monthly ECGs.
[24]	Owen et al.	Single-Center Retrospective Cohort Study	N = 1027	Did not specify	No arrhythmias occurred during or after pregnancy.
[25]	Kubota et al.	Single-Center Retrospective Cohort Study	N = 306	Bisoprolol 1.25-5 mg/day; Atenolol 30-60 mg/day; Metoprolol 120 mg/day; Nadolol 60 mg/day	Neonatal hypoglycemia was more frequent in newborns from mothers administered carvedilol compared to those in the control group.
[26]	Nakatsukasa et al.	Case Report	N = 1	Landiolol; Dexmedetomidine; Lidocaine; Mexiletine; Carvedilol 2.5 mg/day	QTc improvement and resolution of arrhythmia were observed, with an increase in left ventricular ejection fraction (LVEF). Postpartum premature ventricular contractions (PVCs), QT prolongation, TdP, and ventricular fibrillation (VF), required defibrillation and treatment with landiolol, dexmedetomidine, lidocaine, and mexiletine.

Efficacy of Beta-Blockers in Reducing Arrhythmic Events in Pregnant Women With LQTS

A case-control study by Heradien et al. examined 36 women with LQTS to assess the efficacy of beta-blocker therapy in preventing cardiac events during and after pregnancy [14]. The study distinguished between symptomatic and asymptomatic carriers of LQTS, concluding that beta-blocker therapy was highly effective in reducing cardiac events in symptomatic carriers. Women who received beta-blocker treatment experienced significantly fewer cardiac events compared to those who were untreated, underscoring the essential role of beta-blockers in mitigating cardiac risks in this vulnerable population.

Similarly, Kaizer et al. conducted a multicenter retrospective study that reinforced the importance of continuing beta-blocker therapy throughout pregnancy [15]. Their research demonstrated that beta-blockers were crucial in managing cardiac risks in pregnant women with LQTS, further emphasizing the necessity of these medications during pregnancy.

Other studies have also provided valuable insights into the efficacy of beta-blockers during various stages of pregnancy and delivery. For instance, Kamiya et al. reported a case in which a patient with LQTS, treated with beta-blockers, experienced no complications during the peripartum period. This suggests that beta-blockers may not only be effective for LQTS but also for other conditions, like Andersen-Tawil syndrome [16]. Tanaka et al. further observed that beta-blocker use may help prevent cardiovascular events during labor, indicating the protective benefits of beta-blockers during critical phases of pregnancy when the risk of arrhythmic events is elevated [17]. Kubo et al. demonstrated that the use of beta-blockers during labor and delivery was safe and effective, with delivery proceeding without hemodynamic, anesthetic, or obstetric complications in women receiving beta-blockers [18].

Collectively, these findings underscore the importance of beta-blocker therapy for pregnant women with LQTS. The consistent observation across multiple studies that beta-blockers effectively reduce arrhythmic events and contribute to safer pregnancy outcomes strongly supports their continued use in managing LQTS during pregnancy and beyond.

Safety and Risks of Beta-Blocker Therapy for the Fetus

The safety and potential risks associated with beta-blocker therapy during pregnancy have been extensively studied, resulting in a complex understanding of the potential fetal outcomes. Various studies offer insights into the impact of beta-blocker exposure on fetal development, with mixed and sometimes conflicting results.

Albertini et al. found that term births exposed to beta-blockers had significantly lower birth weights (2783 ± 283 g) compared to those without beta-blocker exposure (3084 ± 256 g) [19]. Another case reported by Lee et al. highlighted how the use of nadolol following implantation resulted in 30 previous implantable cardioverter-defibrillator (ICD) shocks. In contrast, midodrine demonstrated better compliance in managing the condition [20]. This study highlighted that atenolol and nadolol were associated with an increased risk of IUGR, emphasizing the need for careful consideration when prescribing these medications during pregnancy. Similarly, Ishibashi et al. conducted a retrospective study involving 76 women and reported a significantly lower birth weight in infants exposed to beta-blockers (2445 ± 613 g) compared to the control group (2914 ± 414 g), further supporting a correlation between beta-blocker exposure and reduced birth weight [8].

The results from these studies suggest that the impact of beta-blockers on fetal development may vary depending on the specific medication used and the clinical context [8,19]. Kaizer et al. found that while nadolol, propranolol, and metoprolol did not negatively affect fetal heart rate in LQTS-negative or LQT3 fetuses, beta-blockers were associated with bradycardia in fetuses with LQT1 and LQT2, respectively. Additionally, atenolol was linked to significant fetal growth restriction, leading to recommendations against its use during pregnancy [15]. This underscores the importance of selecting the appropriate beta-blocker based on the LQTS subtype to minimize fetal risks.

Ersbøll et al. found that beta-blocker use during pregnancy increased the risk of delivering a small gestational age (SGA) infant nearly threefold, even after adjusting for maternal BMI. Their study also noted a higher incidence of congenital malformations in the beta-blocker-exposed group, although this finding was not statistically significant [10]. These results further raise concerns about the potential adverse effects of beta-blockers on fetal development, suggesting that while beta-blockers are essential for managing maternal cardiac risks, careful evaluation of fetal safety is necessary.

In contrast to these findings, Heradien et al. found that fetal distress rates were similar between beta-blocker-treated and control groups, aligning with general population norms. While birth weights were slightly lower in beta-blocker-exposed infants (mean 2900 g), the difference in birth weight was less pronounced compared to findings in other studies [14]. A case reported by Milner et al. described a cesarean delivery at 35 weeks due to IUGR and preeclampsia, illustrating that beta-blocker therapy may occasionally necessitate early delivery due to complications [21].

Additionally, Kubo et al. documented a fetus weighing 2154 g with a heart rate of 130 beats per minute and normal Apgar scores after delivery, suggesting that, despite lower birth weights, some infants may still experience favorable immediate postnatal outcomes [18]. Huttunen et al. observed significant postnatal catch-up growth in infants exposed to beta-blockers in utero, with a growth rate of 0.08 SDS (Standard Deviation Score)/month compared to steady growth of 0.01 SDS/month in those not exposed [22]. This finding indicates that, although beta-blocker exposure may initially result in lower birth weights, affected infants can demonstrate substantial postnatal growth recovery.

In conclusion, these findings reflect the nuanced and sometimes conflicting outcomes regarding the safety and risks of beta-blocker therapy for the fetus. While there is consistent evidence pointing to increased risks, such as lower birth weight and IUGR, other studies highlight minimal adverse effects and the potential for significant postnatal catch-up growth. This emphasizes the need for individualized assessment and management strategies when considering beta-blockers during pregnancy, balancing maternal cardiac health benefits against potential fetal risks.

Postpartum Management of LQTS

Effective postpartum management of LQTS is crucial for minimizing the risk of cardiac events. Multiple studies highlight the importance of continuing beta-blocker therapy during this period. Seth et al. demonstrated that beta-blockers significantly reduce the occurrence of postpartum cardiac events, strongly recommending their continued use [4]. Their research specifically noted that women with LQTS, particularly those with the LQT2 genotype, have a lower risk of cardiac events during pregnancy but experience an elevated risk during the nine-month postpartum period. Beta-blocker therapy reduced the annualized cardiac event rate during this high-risk time from 3.7 events per year to 0.8 events per year. Additionally, a graphical representation in the study showed a marked increase in the likelihood of a first cardiac event within six months postpartum, with the risk gradually decreasing thereafter [4].

Meregalli et al. also emphasized the significance of the postpartum period, noting a substantial rise in cardiac events, particularly among patients with LQT2 [27]. They recommended regular ECG monitoring every one to two weeks postpartum, especially when QT duration is notably prolonged compared to pre-pregnancy values or exceeds 500 ms. This approach enables close observation of patients, aiding in the identification of those at increased risk of adverse cardiac events.

Therefore, frequent ECG monitoring and appropriate adjustments to beta-blocker dosage based on individual clinical responses are essential during the postpartum period. Regular ECGs are particularly important in cases where QT prolongation was observed during pregnancy, as suggested by Seth et al. [4]. These management strategies are vital for mitigating potential cardiac risks and ensuring the safety and well-being of postpartum women with LQTS.

Discussion

Background

LQTS presents significant challenges for pregnant women due to the heightened risk of arrhythmias. Early diagnosis and timely initiation of beta-blocker therapy in high-risk patients have been shown to reduce the occurrence of cardiac events during both pregnancy and the postpartum period. However, the administration of beta-blockers during pregnancy requires careful consideration of potential risks to the fetus, such as IUGR. Therefore, regular fetal growth assessment is essential to monitor for any adverse effects. Close monitoring and individualized treatment plans remain crucial to achieving favorable maternal and fetal outcomes, with continued therapy postpartum to protect against the increased risk of cardiac events during this high-risk period [8,23].

Summary of Findings

This systematic review synthesizes the available evidence from observational studies and case reports on the use of beta-blockers in pregnant women with LQTS. The findings indicate that beta-blockers are effective in significantly reducing arrhythmic events during pregnancy and postpartum. However, potential fetal risks, including decreased birth weight, IUGR, fetal bradycardia, and other neonatal complications, are associated with maternal use of beta-blockers during pregnancy. As a result, careful monitoring of both the mother and fetus is essential, including regular fetal heart rate assessments and maternal ECGs. Additionally, beta-blocker dosages should be adjusted throughout pregnancy, delivery, and the postpartum period based on individual clinical responses. Continued beta-blocker therapy postpartum is essential in preventing cardiac events, particularly during the heightened-risk period following delivery.

Efficacy of Beta-Blockers in Reducing Arrhythmic Events in Pregnant Women With LQTS

The included studies evaluated the safety and efficacy of various beta-blockers, including propranolol, atenolol, metoprolol, nadolol, and carvedilol, in managing LQTS during pregnancy. Multiple studies demonstrated that pregnant women with LQTS who received beta-blocker therapy experienced significantly fewer cardiac events and arrhythmias compared to those who were untreated [8,14,15].

Further support for these findings comes from additional studies by Kamiya et al., Tanaka et al., Kubo et al., Lee et al., and Owen et al., which showed that beta-blockers not only reduce cardiac events during pregnancy but also provide protection during critical periods, such as labor and delivery [16-18,20,24]. These studies collectively suggest that the benefits of beta-blocker use during pregnancy, particularly in reducing arrhythmic events, outweigh their potential risks, making them a key therapeutic option for pregnant women with LQTS.

Safety and Risks of Beta-Blocker Therapy for the Fetus

The conflicting outcomes in the literature regarding the safety of beta-blockers on fetal development highlight the complexity of managing pregnant women with LQTS. Fetal responses to beta-blocker therapy appear to be influenced by both the specific medication used and the underlying maternal conditions. This necessitates a careful balance between optimizing maternal health and mitigating potential risks to the fetus, underlining the need for personalized management strategies.

The studies by Albertini et al. and Ishibashi et al. demonstrated a significant association between beta-blocker exposure, particularly with atenolol and nadolol, and adverse fetal outcomes, such as lower birth weights and an increased risk of IUGR [8,19]. These findings underscore the importance of selecting the appropriate beta-blocker, especially in women at risk for fetal growth complications. On the other hand, research by Huttunen et al. showed that infants exposed to maternal beta-blockers, particularly LQT1 patients, exhibited significant postnatal catch-up growth despite being smaller at birth, suggesting that some adverse effects may be reversible postnatally [22].

Another concern that has emerged from the literature is the increased risk of SGA infants, as reported by Ersbøll et al., although this study did not find a significant rise in congenital malformations [10]. Fetal bradycardia, especially in LQT1 and LQT2 fetuses, has also been reported with medications such as nadolol, propranolol, and metoprolol, further complicating the choice of beta-blocker in pregnant women with LQTS [15]. Additionally, concerns about neonatal hypoglycemia, particularly with carvedilol use, have been raised, with some studies suggesting a higher incidence compared to control groups [25].

However, it is important to note that not all studies reported negative fetal outcomes. Research by Papantoniou et al., Kamiya et al., and Kubo et al. reported smooth deliveries with normal fetal vitals and cardiac function despite maternal beta-blocker use [16,18,23]. These findings suggest that, under appropriate management, beta-blocker therapy may not universally lead to adverse fetal outcomes and may, in fact, be safe for many patients.

Overall, the data suggest that while beta-blockers play a critical role in managing maternal cardiac risks, their use during pregnancy must be approached with caution. A personalized treatment plan that includes regular fetal monitoring is crucial to ensure that maternal benefits are not outweighed by fetal risks. These findings underscore the need for further research to refine treatment guidelines for pregnant women with LQTS.

Clinical Guidelines and Recommendations

According to the former FDA pregnancy classification system, beta-blockers such as bisoprolol, carvedilol, labetalol, metoprolol, nadolol, and propranolol were classified as Category C, meaning no well-controlled studies had been conducted in humans, though animal studies suggested potential adverse effects on the fetus. Atenolol, however, was classified as Category D, indicating evidence of human fetal risk; though, in certain clinical situations, the benefits may outweigh these risks. The 2018 ESC guidelines specify that beta-blockers, including bisoprolol and propranolol, are associated with fetal bradycardia and hypoglycemia. These guidelines further caution against the use of atenolol during pregnancy due to its heightened risk of fetal growth restriction [15].

Several studies have formulated effective clinical protocols and recommendations aimed at optimizing both maternal and fetal health. Central to these guidelines is the administration of beta-blockers during pregnancy for mothers with LQTS, with the continuation of therapy throughout the postpartum period to prevent potential cardiac events. Although the benefits of beta-blockers are well documented, their use requires careful consideration of fetal risks and the mother’s specific clinical condition.

Monitoring and Management Strategies

A variety of strategies are employed to monitor maternal and fetal well-being during beta-blocker therapy. These include regular high-risk pregnancy ECGs for the mother, fetal echocardiography to assess fetal arrhythmias and cardiac structure, ultrasound scans to monitor fetal growth, and biophysical profiles to evaluate overall fetal health. These tools are essential for ensuring that maternal cardiac conditions are managed appropriately while minimizing the risk of fetal complications throughout pregnancy and the postpartum period.

Postpartum Management of LQTS

Effective postpartum management of LQTS, particularly through continued beta-blocker therapy, is crucial for reducing the risk of cardiac arrhythmias, as several studies have shown [21,26]. The risk of cardiac events is highest within the first six months after childbirth, especially in patients with the LQT2 genotype [4,14]. Thus, close monitoring and adjustment of beta-blocker dosages, based on individual response and clinical status during this period, are essential. Recommendations include frequent ECGs during the early postpartum phase and careful observation, particularly when QT duration is significantly prolonged compared to pre-pregnancy values, or when QT intervals exceed 500 ms [27].

Comparison With Existing Literature

Our findings align with existing systematic reviews that demonstrate the efficacy of beta-blockers in reducing cardiac events and arrhythmias in women with LQTS during pregnancy and the postpartum period. However, our review provides added value by including recent studies, allowing for a more contemporary analysis of the safety and efficacy of beta-blocker therapy. Our review expands on existing literature by incorporating the latest evidence on the variability of fetal risks, emphasizing the importance of tailoring treatment strategies based on the LQTS genotype and the specific beta-blocker used. While previous studies have noted potential risks, such as reduced birth weight or IUGR, our review delves deeper into the impact of different medications and clinical contexts.

Limitations

Despite our comprehensive search strategy, there remains a possibility of reporting bias, as some relevant studies may not have been captured by our predefined search criteria. Additionally, as with all systematic reviews, our study is constrained by the variability across individual studies, including differences in trial design, sample sizes, and follow-up durations, which may affect the generalizability of our findings. Another limitation is the exclusion of studies where the full text was unavailable. Although this decision was made to ensure a thorough assessment of study quality and risk of bias, it may have resulted in the omission of potentially valuable data from abstract-only studies. Finally, the lack of uniformity in the reporting of outcomes, particularly regarding fetal safety, could influence the strength of the conclusions drawn. Nonetheless, our review provides a comprehensive and up-to-date analysis of the role of beta-blockers in reducing cardiac events in pregnant women with LQTS and highlights areas where further research is needed.

Future potential studies

Future research should focus on the long-term outcomes of children exposed to beta-blockers in utero, particularly examining developmental milestones and the potential for late-onset adverse effects. Understanding the broader childhood health implications of prenatal beta-blocker exposure is essential for providing a more comprehensive assessment of its safety. Additionally, studies should aim to refine personalized beta-blocker dosing regimens for pregnant women with LQTS, ensuring that treatment is tailored to optimize both maternal and fetal safety. Further investigation is also necessary to develop robust postpartum care plans for women with LQTS, emphasizing continuous monitoring and management during this particularly high-risk period. These future studies would help bridge existing knowledge gaps and improve clinical guidelines for the management of LQTS in pregnant women.

## Conclusions

In conclusion, the management of LQTS in pregnant women through beta-blocker therapy offers significant benefits, particularly in reducing arrhythmic events during pregnancy and the postpartum period, thereby ensuring maternal cardiac safety. However, this review also highlights the potential fetal risks associated with beta-blocker use, such as lower birth weight, fetal bradycardia, and an increased risk of IUGR. These findings underscore the need for individualized treatment plans that balance maternal health benefits with fetal safety, along with regular monitoring and dose adjustments to optimize outcomes for both the mother and fetus.

## Appendices

Quality and risk of bias assessment

We conducted a comprehensive methodological quality assessment to ensure the reliability and trustworthiness of the evidence included in our systematic review. Randomized Controlled Trials (RCTs) were assessed using the Cochrane Risk of Bias Tool, which evaluates five domains of bias: (a) bias arising from the randomization process; (b) bias due to deviations from intended interventions; (c) bias caused by missing outcome data; (d) bias in outcome measurement; and (e) bias in the selection of reported results.

For cohort and case-control studies, we applied the Newcastle-Ottawa Scale (NOS), which assesses three domains: selection (four items), comparability (one item), and outcome/exposure (three items). A study was classified as Good quality if it scored three or four stars in the selection domain, one or two stars in the comparability domain, and two or three stars in the outcome/exposure domain. It was classified as Fair quality if it scored two stars in the selection domain, one or two stars in the comparability domain, and two or three stars in the outcome/exposure domain. Studies were rated as Poor quality if they scored zero or one star in the selection domain, zero stars in the comparability domain, or zero or one star in the outcome/exposure domain.

For case reports, we utilized a checklist developed based on the CARE guidelines [13]. This checklist comprised eight domains: (1) Patient Information: a comprehensive description of patient demographics, medical history, and clinical findings; (2) Clinical Findings: transparent reporting of clinical examinations and diagnostic test results; (3) Timeline: an adequate and precise timeline of events; (4) Diagnostic Assessment: an accurate description of diagnostic criteria and methods; (5) Therapeutic Interventions: a detailed description of interventions, dosages, and duration; (6) Follow-Up and Outcomes: an appropriate follow-up period with clear reporting of outcomes; (7) Discussion: comparison with existing literature and implications for clinical practice; and (8) Informed Consent: a statement confirming that informed consent was obtained from the patient. Each domain was rated as "Yes," "No," or "Insufficient."

Quality assessment of included studies

Quality Assessment of the Observational Studies

In total, nine observational studies were included in our review. The Selection scores ranged from one to four stars, with most studies receiving three or four stars, indicating generally robust selection methods. Comparability was often less well addressed, with several studies receiving no stars due to insufficient control for confounding variables. The Outcome scores varied, with most studies receiving two or three stars, reflecting adequate follow-up and outcome measurement.

Overall, the risk of bias was deemed "Good" for the majority of the studies, with only a few categorized as "Fair" or "Poor." This suggests that, while the studies generally employed strong selection methods and reliable outcome measurements, confounding variables were not consistently well-controlled across all studies. The results of the quality assessment of observational studies, conducted using the NOS, are presented in Table 3.

**Table 3:** Quality assessment of observational studies using Newcastle-Ottawa Scale (NOS) "Selection" assesses the quality of participant selection methods, focusing on representativeness and the initial absence of the outcome. "Comparability" evaluates control for confounding factors but is not rated in this assessment due to the uncontrolled nature of the studies. "Outcome" rates the measurement and reporting clarity of the outcomes of interest.

Study references	Selection (max 4 stars)	Comparability (max 2 stars)	Outcome (max 3 stars)	Overall risk of bias
[22]	2 Stars	-	2 Stars	Fair
[10]	3 Stars	1 Star	3 Stars	Good
[24]	1 Star	-	1 Star	Poor
[15]	4 Stars	2 Stars	3 Stars	Good
[4]	4 Stars	1 Star	3 Stars	Good
[25]	4 Stars	1 Star	2 Stars	Good
[8]	4 Stars	1 Star	3 Stars	Good
[19]	4 Stars	1 Star	3 Stars	Good
[17]	3 Stars	1 Star	3 Stars	Good

Quality Assessment of the Included Case Reports

In total, eight case studies were included in our review. Each element is marked as "Yes" (Y), "No" (N), or "Insufficient" (I). Most case studies provided comprehensive patient information, clinical findings, and therapeutic interventions. However, some studies lacked sufficient detail in diagnostic assessment and informed consent documentation. The results of the quality assessment for case reports, using the simplified CARE checklist, are presented in Table 4.

**Table 4:** Quality assessment of case studies using the CARE guidelines Y: Yes; N: No; I: Insufficient

Study references	Patient information	Clinical findings	Timeline	Diagnostic assessment	Therapeutic intervention	Follow-up and outcomes	Discussion	Informed consent
[21]	Y	Y	Y	Y	Y	Y	Y	Y
[23]	Y	Y	Y	Y	Y	Y	Y	I
[26]	Y	Y	Y	Y	Y	Y	Y	Y
[16]	Y	Y	Y	Y	Y	Y	Y	I
[24]	Y	Y	Y	Y	Y	Y	Y	I
[7]	Y	Y	Y	Y	Y	Y	Y	I
[18]	Y	Y	I	I	Y	I	Y	N
[14]	Y	Y	Y	Y	Y	Y	Y	Y
